# Mapping Risk of Malaria Transmission in Mainland Portugal Using a Mathematical Modelling Approach

**DOI:** 10.1371/journal.pone.0164788

**Published:** 2016-11-04

**Authors:** Eduardo Gomes, César Capinha, Jorge Rocha, Carla Sousa

**Affiliations:** 1 Centro de Estudos Geográficos, Instituto de Geografia e Ordenamento do Território, Universidade de Lisboa, Lisboa, Portugal; 2 CIBIO/InBIO, Centro de Investigação em Biodiversidade e Recursos Genéticos, Cátedra Infraestruturas de Portugal-Biodiversidade, Universidade do Porto, Porto, Portugal; 3 Zoologisches Forschungsmuseum Alexander Koenig, Bonn, Germany; 4 Global Health and Tropical Medicine, GHTM, Instituto de Higiene e Medicina Tropical, Universidade Nova de Lisboa, Lisboa, Portugal; Universidade Federal de Minas Gerais, BRAZIL

## Abstract

Malaria is currently one of the world´s major health problems. About a half-million deaths are recorded every year. In Portugal, malaria cases were significantly high until the end of the 1950s but the disease was considered eliminated in 1973. In the past few years, endemic malaria cases have been recorded in some European countries. With the increasing human mobility from countries with endemic malaria to Portugal, there is concern about the resurgence of this disease in the country. Here, we model and map the risk of malaria transmission for mainland Portugal, considering 3 different scenarios of existing imported infections. This risk assessment resulted from entomological studies on *An*. *atroparvus*, the only known mosquito capable of transmitting malaria in the study area. We used the malariogenic potential (determined by receptivity, infectivity and vulnerability) applied over geospatial data sets to estimate spatial variation in malaria risk. The results suggest that the risk exists, and the hotspots are concentrated in the northeast region of the country and in the upper and lower Alentejo regions.

## Introduction

Malaria is currently an endemic disease in about 100 countries, namely in tropical and sub-tropical regions [1]. Around 80% of all malaria cases occur in Africa. In 2015, 3.2 billion people were at risk and it is estimated that there were 214 million cases [1]. Every year, there are about a half-million deaths, most of them children less than 5 years old [1].

Malaria in humans is caused by 5 species of protozoans of the genus *Plasmodium* [2,3]: *Plasmodium falciparum*; *Plasmodium ovale*; *Plasmodium vivax*; *Plasmodium malariae*; and *Plasmodium knowlesi* [4]. In order for humans to become infected, the protozoans need to be transmitted through the bite of an infected female mosquito of the *Anopheles* genus.

In the European continent, malaria was eradicated from many countries in the past as a consequence of targeted prevention and control measures (*e*.*g*. application of DDT—dichlorodiphenyltrichloroethane), habitat modifications and a general improvement human living standards. However, apart the notorious absence of cases in 2015, indigenous infections have been occurring in Europe in recent years, mainly in Eastern and Southern Europe [5].

In Portugal, the status of malaria before the 1950s is well documented in the studies of Francisco Cambournac. This author identified 6 malariologic regions. These regions revealed different levels of endemicity, which are largely associated with the suitability of habitat to the vector mosquito *An*. *atroparvus* [6,7]. In 1973, after an intensive work of the national authorities, the indigenous strains of malaria parasites were considered eradicated from Portugal by the World Health Organization (WHO).

After several decades of endemic malaria eradication, some European countries such as Italy, Germany, Spain and France, have reported cases of autochthonous transmission [8]. More recently, in 2012, Greece faced its first outbreak of malaria in this century. Therefore, despite the absence of indigenous cases of transmission in 2015, the possibility of resurgence of malaria outbreaks in Europe remains [5].

Entomological studies on *An*. *atroparvus*, a widely dispersed native mosquito, have demonstrated that it has competence (albeit, low) for transmitting imported strains of *P*. *falciparum* [9]. Moreover, due to strong historical and cultural affinities, Portugal has a close relationship with an important number of malaria-endemic countries such as Angola, Mozambique or Brazil [10]. Travellers arriving from these regions provide a regular number of imported infections, ultimately contributing for the risk of indigenous malaria transmission.

In this context, more than four decades after the last autochthonous case in Portugal, it is relevant to study the possibility of resurgence of indigenous malaria outbreaks in Portugal.

The aim of this study was to analyse the malariogenic potential of Portugal according to the parameters of receptivity, infectivity and vulnerability (e.g. [11,12]). Here we present the first model for the risk of resurgence of malaria in mainland Portugal. The model used entomological parameters and geospatial data representing factors for *An*. *atroparvus* and geospatial data [9]. The resulting model allows us to assess in a semi-qualitative way the risk of malaria resurgence.

We have built 3 different scenarios: (a) scenario A0—current risk of malaria transmission (with the malaria patients diagnosed by DGS—Direção-Geral da Saúde); (b) scenario A1—risk of malaria (with 200 infected people); (c) scenario A2—risk of malaria (with 400 infected people).

All the procedures and analysis were carried out in a Geographic Information System (GIS) environment. The data were classified using an ETRS89/PT-TM06 projection system using ESRI ArcGIS 10.3 software. The aim of implementing GIS was to provide a standardised, verified set of core data that could be easily accessed to support malaria research and control [13].

## Materials and Methods

To assess the risk of malaria resurgence, we used the malariogenic potential that consists of the product of three components: 1. receptivity (or vector capacity); 2. infectivity (or vector competence); and 3. vulnerability (imported cases of malaria). We describe how we obtained each of the three factors below.

### Receptivity

The receptivity, determined according to the vectorial capacity (*C*) and expresses the ability of the vector species to transmit the pathogen. This index, designed by [14], is one of the major tools used in malaria epidemiology [15]. The *C* value assesses the number of future infections that will result from one currently infective case of malaria [16]. This index is estimated by the following equation:

$$C=\frac{{ma}^{2}p^{n}}{-ln\;p}$$ (1)

where *ma* is the biting rate (*m* is the relative density of mosquitoes in relation to human density and *a* the average number of persons bitten by one mosquito in one day), *p* is the proportion of mosquitoes surviving per day, and *n* represents the parasite’s extrinsic incubation period or extrinsic cycle in days.

#### Biting rate estimation

The biting rate translates the average number of mosquitoes that bite humans daily. It can be estimated as the relative density of mosquitoes in relation to human density (*m*) multiplied by the average number of bites that a single person experiments during one day (a- man biting habit). This parameter was estimated directly from all-night human-landing collections made in Comporta region, a former malaria’s hiperendemic area where *P*. *falciparum* was the dominant parasite species. Based on a worst-scenario case this estimates was extrapolated for the whole of Portugal. Man-biting habit calculation (a) was achieved through the product of *An*. *atroparvus* biting frequency (*f*) plus the anthropophilic index of the species (HBI). While *f* was evaluated based on the feeding frequency (per day) of laboratory reared females to which was given the daily possibility of feeding on an appropriated host, HBI was assessed by an ELISA-two sites performed with the mid-gut content of freshly-fed females [9]. All biological material (larvae and females) were collected in Comporta region following the same rationale mentioned previously.

#### Distribution data, abundance, and suitability of *An*. *Atroparvus*

Between 2001 and 2003, numerous field surveys covering several localities along mainland Portugal were made to collect adult mosquitoes in human and animal facilities by means of electrical aspirators. The diverse surroundings of the surveyed places led to highly irregular abundance values, even between close sites. Therefore, a low abundance level does not necessarily imply that the area is less suitable. However, the opposite holds true, places with high abundance are environmentally suitable.

Moreover, the survey only gives us abundance records of the whole *An*. *maculipennis* complex because there is a high inter-species similarity between adult *Anopheles* mosquitoes.

*An*. *atroparvus* is the only one of the complex that can be found in the Algarve [17] and Alentejo [18] and highly prevails in central Portugal (9 to 1 proportion) [19] and Serra da Estrela Natural Park (6 to 1 proportion) [20]. Other existing studies report a majority abundance of *An*. *atroparvus*, although they do not advance quantitative data (e.g. [20,21]).

With this information, we have managed to obtain a general abundance ratio of eight *An*. *atroparvus* to one *An*. *maculipennis*. Still, because even these higher abundance values are possibly biased due to the unevenness of the surveyed facilities, they were transformed into ‘presence only’ records. Absences too were obtained. However, only the sites where other mosquito species were present but *An*. *maculipennis* complex had null/reduced values of abundance (<2%) were considered. This allowed us to remove false absences due to mosquito mitigation actions and/or facility specific characteristics. A total of 76 presence and 16 absence records were referenced.

We conducted entomological surveys in 92 locations in well-known mosquito habitats (e.g. stables, pig and cattle shelters, and other animal hutches).

Data from all collection sites associated with mosquito development, were gathered [7]. Determining potential predictive variables among the factors that affect the distribution of the species is crucial to model potential habitats [22]. Knowing the significant ecological factors that play a relevant role in the distribution of the species is important to integrate independent variables into predictive models.

#### Environmental factors

The selection of the environmental data with the uppermost predictive value was based on both prior knowledge of the prevailing factors in the distribution of *An*. *atroparvus* and data availability. Therefore, five environmental factors were selected [7], namely: i) mean maximum temperature of the warmest quarter; ii) mean minimum temperature of the coldest quarter; iii) mean total annual precipitation; iv) wetland density and suitability index; and v) agricultural density and suitability index.

The temperature is included because it has direct influence on the species’ physiology and behaviour [23] or indirect effect on the access to larval sites and on the depth variability of the water bodies. Using the mean maximum temperature of the warmest quarter and the mean minimum temperature of the coldest quarter together to represent a single ecological factor avoids the spatial and temporal homogenization that comes from the application of a unique annual mean model.

The precipitation is correlated with the productivity of the mosquito breeding sites. This is expressed on [24]. The precipitation was taken into account due to the strong effects on the productivity of the *Anopheles’* breeding sites. Precipitation is highly correlated with the availability and characteristics (i.e. depth of the water bodies and the existence of temporary ponds) of *An*. *atroparvus’* habitats in the aquatic phase of its life cycle [24]. Both temperature and precipitation datasets were downloaded in raster format (1km^2^ grid) from the WORLDCLIM project (gridded climate data) [25]. The time period of the climatological records is from 1950 to 2000. This time period expresses the climatological variability occurred in Portugal in the last decades [26].

To include data on the abundance and closeness of the wetlands, which have a direct relationship with the aquatic life cycle of *An*. *atroparvus* [27], we have used the Corine Land Cover 2006 (CLC2006) spatial raster dataset for Portugal (the resolution of the data is 100 x 100 meters) which is produced by the European Environment Agency (EEA) (available at: http://www.eea.europa.eu/data-and-maps/data/corine-land-cover-2006-raster-3) [28]. The wetland density and suitability index; and agricultural density and suitability index was based on land use classes wetland and agricultural areas, respectively, by calculating a buffer with 10 km radius. We attributed different weights to each wetland category taking into account its known suitability for larval development of *An*. *atroparvus*. The categories that were taken into account were rice fields, inland marshes, large inland water bodies, estuaries, coastal lagoons, intertidal flats, permanently irrigated areas, watercourses, and salt pans. The final index results from the product of the density values and the factors used for weighting.

Finally, the agricultural density and suitability index was addressed as an indicator of highest *An*. *atroparvus* larval productivity [29]. For instance, due to the zoophilic nature of *An*. *atroparvus* places such as cattle farms have a high suitability for the development of large populations [9]. And because of this, we have assumed that areas characterized by a high agricultural intensity have a greater probability of containing cattle. This index comprised the following classes derived from the CLC2006s nomenclature: pastures, non-irrigated arable land, permanently irrigated land, rice fields, vineyards, fruit trees and berry plantations, olive groves, annual crops associated with permanent crops, complex cultivation patterns, land principally occupied by agriculture, with significant areas of natural vegetation and agro-forestry areas [28]. The final calculation was obtained in the same way as the wetland index.

#### Algorithms used for modelling environmental suitability

For the suitability model for *An*. *atroparvus*, we have used five different algorithms (they are the most commonly used in recent studies), three of which had already been tested in previous experiments [7], i.e. Logistic Regression, Mahalanobis distance (D2), and Artificial Neural Networks (ANN), plus two added for this study, Maximum Entropy and a Genetic Algorithm.

The majority of the predictive models working on presence/absence data are derived from well-established statistical techniques [30]. One good examples of this type of approach is the use of logistic regression that does not require the normality assumption of independent variables and is more robust when the same is not met. These models are based on the existence of a simple function for the relationship between the presence/absence of species and a set of environmental variables [31]. These strategies can produce realistic and simple models for this high function interpretability in understanding natural processes.

All the models based in presence/absence datasets can be used with pseudo absences. The use of pseudo absences can include an error rate in the model because it can include suitability areas as ‘false zeros’[32]. However, the absence of evidence is not an evidence of absence. Absence records can be due only to chance.

For the models that were initially designed for only data of presence, the best way to sort them is in relation to the degree of complexity in the processes involved. The methods of distance (or similarity environmental models) are simple representations of the ecological niche. They are based on the existence of an optimum ecological point for each species defined by the central feature of the presence points in the ecological space.

Mahalanobis distance generates an ellipsoidal envelope around the optimum point of the ecological space. Mahalanobis distance includes greater complexity than simple Euclidean distance, because it takes into account the covariance matrix between the environmental variables in the occurrence. This allows to interpret the model as an expression of the environmental constraints that the species suffers including the correlations between variables, but requires a number of sample points greater than the number of environmental variables [33].

The Maxent (Maximum Entropy) starts a list of more complex models. This is a machine learning that estimates the probability distribution closer to uniform distribution under the restriction that the expected values for each environmental variable should follow the empirical values observed in the presences. Similarly to the logistic regression, the MaxEnt weighs each variable with a constant. The principle of maximum entropy indicates that the distribution model that satisfies all constraints should be as uniform as possible, allowing to make predictions from incomplete information [34].

Artificial neural networks (ANN) and GARP (genetic algorithm for rule set production). Both share a lot of theoretical structure common to auto-learning methods, i.e. artificial intelligence. The ANN are computational models inspired by the central nervous system that are able to perform machine learning processes. They are generally presented as systems of interconnected neurons that can compute, approximate and even incorrect, quantitative data, with a gradual degradation of the response. ANN have a great power of representation of knowledge through the creation of relationships between the weighted system entries but difficulty to turn explicit the knowledge acquired by the network through a language understandable by humans, i.e. black box models [35].

Finally, the GARP represents a hybrid technique that includes statistical techniques (logistic regression) and bioclimatic envelopes within a machine learning strategy. GARP is not a modelling technique for presence data because the adjustment is done by generating a set of pseudo absences. Though, it employs sophisticated techniques to treat this problem [36].

#### Combination of models

In order to calibrate each model the records dataset of *An*. *atroparvus* was split into both calibration (called the training set) and validation (or testing set) datasets. For that, we use a k-fold cross-validation, with the original sample randomly portioned into k = 3 equal sized subsamples. This method avoids the unnecessary use of records for validation, performing better than a unique validation set, when only a small amount of records (n = 92 in this case) exists [37].

Hence, three calibration/validation sets were created, each one of them composed of 13 records (2 absences and 11 presences). This value matches a random extraction by around 15% for each record type, permitting the prevalence of the species to remain unaffected in all the calibration sets. The advantage of this method is that all observations are used for both training and validation. Furthermore, each observation is only used once for validation.

The kappa index [38] was used as predictive evaluation measure. This index demonstrates the agreement between the correct classifications achieved and the expected by chance. Its values vary from 0 and 1. The value 1 corresponds to a perfect agreement between actual and obtained results. A common approach is to set a range of thresholds and calculate the corresponding kappa value for each one of them [39], and then, using the maximum kappa value as the predictive performance of every single model. The five predictive models were partitioned in 20 intervals of equal prediction amplitude (0.05) and the kappa value calculated for each one of the intervals (Table 1).

**Table 1:** % maximum *kappa* of the five predictive models.

Limit	ANN	LR	DM	GARP	ME
**0.05**	-0.05	0.25	0.42	0.51	0.00
**0.10**	0.09	0.44	0.39	0.51	0.44
**0.15**	0.09	0.59	0.00	0.36	0.59
**0.20**	0.39	0.72	0.00	0.31	0.72
**0.25**	0.51	0.72	0.00	0.31	0.57
**0.30**	0.51	0.72	0.00	0.31	0.68
**0.35**	0.51	0.72	0.00	0.31	0.64
**0.40**	0.51	0.64	0.00	0.26	0.50
**0.45**	0.51	0.75	0.00	0.31	0.31
**0.50**	0.51	0.75	0.00	0.31	0.16
**0.55**	0.51	0.75	0.00	0.31	0.11
**0.60**	0.51	0.75	0.00	0.31	0.07
**0.65**	0.51	0.77	0.00	0.31	0.04
**0.70**	0.46	0.77	0.00	0.28	0.04
**0.75**	0.46	0.75	0.00	0.28	0.03
**0.80**	0.43	0.70	0.00	0.28	0.03
**0.85**	0.28	0.55	0.00	0.28	0.03
**0.90**	0.14	0.43	0.00	0.26	0.02
**0.95**	0.05	0.20	0.00	0.11	0.00
**1.00**	0.01	0.10	0.00	0.00	0.00

The results of the *kappa* index for each model demonstrate its predictive capability. For logistic regression, the maximum *kappa* was 0.77 and the maximum entropy 0.72. These values are considered ‘excellent’ [40]. The artificial neural network and genetic algorithm had a *kappa* index of 0.51 and a maximum Mahalanobis distance of 0.42.

For the combination of models we sum all five models weight by the normalized value of their maximum kappa value. The simultaneous use of several combined models has been used as a way to decrease the uncertainty [41]. However, depending on the method used, the final result (i.e. combined model) can either benefit from most accurate models [42]. [43] produced a comparative analysis of several strategies and argued that simple means tend to produce more robust solutions, while other studies point to weighted means approaches [44–46].

#### Estimation of *m* value

The mosquito abundance model used to estimate *m* was computed from this combined suitability map resulting from previous section. Another possibility was to use geostatistical techniques, such as co-Kriging, to retrieve abundance by interpolating directly from the environmental variables. Recent attempts using this technique [47] have only performed fairly R^2^ = 0.58. Therefore, we chose to test two alternative methodologies.

First, based on the final predictive model of habitat suitability, a linear rescheduling [48] of suitability levels for *An*. *atroparvus* was undertaken using the abundance values of this species in different locations in mainland Portugal.

The maximum value of abundance was determined as the arithmetic mean of the 10 highest values of *An*. *atroparvus’* abundance as recorded in field collections. In an attempt to achieve a better correlation between the abundance model and the actual species’ distribution, we tested two different models: 1) the values of the combined model of habitat suitability rescaled on the basis of the average of the 10 highest abundance field survey records; 2) the average value of the 10 lowest and 10 highest abundance records with a linear abundance estimation using the combined model of suitability. In the first test, we rearranged (Eq 2) the resulting values of the suitability combined model (*S*_*cm*_) according to the average (342.7) of the 10 highest abundance records gathered in field surveys (Table 2). Based on this estimate, we obtained an abundance predictive model (*A*^1^_*pm*_) with values between 51 and 321 mosquitoes.

$$A^{1}{}_{pm}={342.7S}_{cm}$$ (2)

**Table 2:** Maximum and minimum values of *An*. *atroparvus’* abundance in mainland Portugal.

Location	Higher Abundance (number of *An*. *atroparvus*)	Suitability	Location	Lower Abundance	Suitability
Quinta de Tourelos	245	0.81	Quinta do Quedeiço	64.3	0.84
Herdade Camarate	255.9	0.89	Tocha	67.2	0.26
Lagoa	274	0.92	Lamas de Orelhão	69.8	0.68
Santo Estevão	303.5	0.84	Alhadas	76.8	0.34
Reguengos Monsaraz	304	0.86	Pechão	78	0.92
Monte da Malhada	342	0.86	Monte do Panasco	78.8	0.69
Comporta	346.7	0.86	São Marcos da Serra	81	0.83
Pego do Altar	348	0.90	Fonte da M.ª Gins	81.9	0.68
Mato Pinheiro	480	0.92	Alagoas	82.1	0.66
Lameiras	528	0.91	S. Bartolomeu Messines	84	0.86
	Mean = 342.7	Mean = 0.87		Mean = 76.4	Mean = 0.68

**Fig 1:**
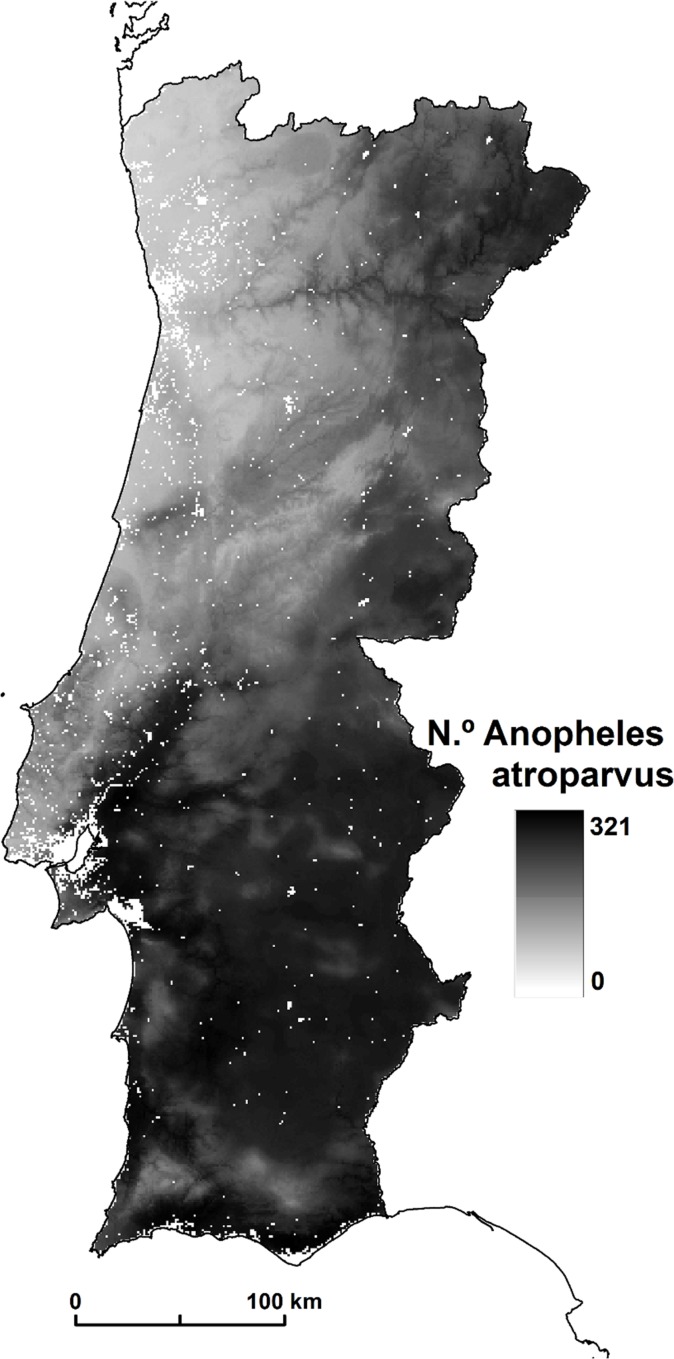
*An*. *atroparvus’* abundance.

Second, in an attempt to evaluate other abundance predictive models, a second scenario was tested (*A*^2^_*pm*_). This consisted in calculating average values from the 10 sites with the highest and lowest values of abundance taken from the combined model of suitability (Table 2). With these two values and the corresponding suitability averages, we performed a linear regression (Eq 3) to predict abundance from the combined models of suitability (Fig 1).

$$A^{2}{}_{pm}={1344.9S}_{cm}-835.48$$ (3)

From these two scenarios, we chose the one with the lowest root mean square error (RMSE). This estimate was based on the differences between the abundance field values and their counterparts in each of the two models. This method showed that the first model performed better, with an RMSE of 141.3, as compared with 146 in the second model.

The model was completed after excluding urban areas, classes represented in the Corine Land Cover [49]. Based on *An*. *atroparvus’* bionomics, these areas were not considered highly suitable habitats for mosquitoes. This species is mainly zoophilic, with a marked preference to feed in livestock and to rest inside animal shelters [6].

The urban land cover classes were distinguished from the Corine Land Cover 2006 [49] classes referenced as ‘Artificial surfaces’. However, the so-called ‘Mine, dump and construction sites’ were not taken into consideration because they would not be attractive to *An*. *atroparvus*; and the class ‘Sport and leisure facilities’ was included only when contiguous to the other selected classes (Table 3).

**Table 3:** Corine Land Cover 2006 classes.

CODE#	LABEL1	LABEL2	LABEL3
**111**	Artificial surfaces	Urban fabric	Continuous urban fabric
**112**			Discontinuous urban fabric
**121**		Industrial, commercial and transport units	Industrial or commercial units
**122**			Road and rail networks and associated land
**123**			Port areas
**124**			Airports
**131**		Mine, dump and construction sites	Mineral extraction sites
**132**			Dump sites
**133**			Construction sites
**141**		Artificial, non-agricultural Vegetated areas	Green urban areas
**142**			Sport and leisure facilities

This information was converted into a raster structure of 1 km^2^ and applied as an urban binary mask (*U*_*mask*_), acting as a process of excluding areas for *An*. *atroparvus’* presence (*A*^1^_*pm*_). Based on Eq 4, we developed a spatially continuous predictive model of abundance (*A*_*map*_), where urban areas correspond to species-refractory areas.

$$A_{map}=U_{mask}{A^{1}}_{pm}$$ (4)

Due to its linear estimation background over the combined model of habitat suitability, the abundance map reveals an identical spatial pattern showing a mosquito variation between 0 and 321 (Fig 1).

**Fig 2:**
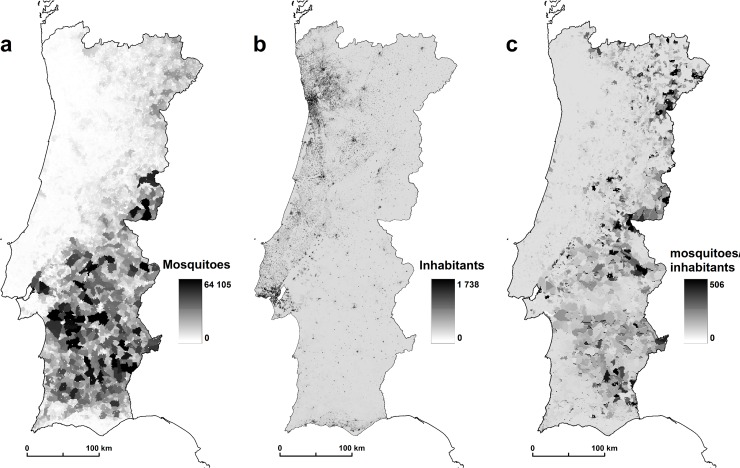
(a) Mosquitoes by subsection, (b) inhabitants by subsection (2011), (c) mosquitoes/inhabitants rate.

To determine the final *m* value, predicted abundance estimates of the vector species were divided by human densities determined through the number of residents by subsection (Fig 2), i.e. urban quarter. Resident population by subsection is produced by Statistics Portugal [50] available at http://mapas.ine.pt/download/index2011.phtml (converted into raster format: 1 km^2^)

#### Biting habit estimation

The biting habit (*a*) corresponds to the product of mosquito biting frequency (number of times a female feeds per day) multiplied by the species human blood index (proportion of freshly-fed females found to contain human blood). Biting frequency was estimated using a group of 29 females that had a daily opportunity to blood-feed on a host. This parameter was obtained by dividing the total number of feeds taken by all females by the sum of days they live. The human blood index is the proportion of recently blood-fed females containing human blood. This index was estimated from a sample of 671 females captured in field collections according to the method of [9]. All 671 blood meals were tested by an ELISA-two sites for the presence of human IgGs.

#### Daily survival rate estimation

The daily survival rate (*p*) can be computed as the *i*_*0*_ root of the proportion of parous females, where *i*_*0*_ is the period in days of the first gonotrophic cycle [51]. The proportion of parous females in the *An*. *atroparvus* population was determined by the ratio between the number of parous females and the number of field-collected females dissected. A parous rate of 0.9 was determined for the natural population during August, the most favourable month for malaria transmission. For the first gonotrophic cycle, this parameter was estimated by Sousa (2008) as 8.5 days. This value was determined with colony specimens (29 females) by averaging the number of days between the emergence of females and their first oviposition.

#### Duration of sporogonic cycle estimation

The duration of the sporogonic cycle in days (*n*) refers to the incubation period of the parasite inside the vector [52]. The time necessary for the extrinsic incubation period to take place depends on both parasite species and temperature (Lysenko and Levitanskaya, 1952; Pavlova, 1952, cited by [53].To study this entomological component we simulated three sporogonic cycle scenarios (in days), one for each type of plasmodium identified when malaria was endemic in Portugal, i.e. *Plasmodium falciparum*, *Plasmodium vivax*, and *Plasmodium malariae* (6). The *n* calculation was based on the method proposed by Moshkovsky [53], according to the following equation:

$$n=T/(t_{a}-t_{m})$$ (5)

Here, *n* is the period in days of the extrinsic cycle of *Plasmodia* and *T* and *t*_*m*_ are constants for each species of human parasite, assuming the values of (*T* = 111; *t*_*m*_ = 16), (*T* = 105; *tm* = 14.5) and (*T* = 144; *tm* = 16) for *P*. *falciparum*, *P*. *vivax*, and *P*. *malariae*, in this order. The variable *t*_*a*_ refers to the average ambient temperature for the insect during the maturation phase.

**Fig 3:**
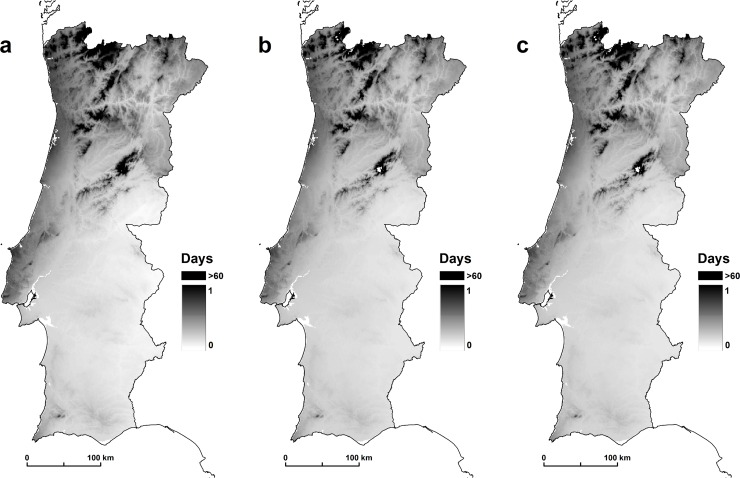
(a) *Plasmodium vivax*, (b) *Plasmodium falciparum*, and (c) *Plasmodium malariae*.

Similarly to *p*, in this study, *n* estimates were computed only for August. This information was expressed in a continuous spatial model (Fig 3) using mean temperature values for the period 1950–2000 (WorldClim project) and assuming, according to the Moshkovsky method [53], that the parasites do not develop below 16°C.

According to [6], it is not plausible that the sporogonic cycle extends beyond 60 days in low temperatures. The optimum number of days for the development of *Plasmodia* at an average temperature of 24°C is 10 days for *Plasmodium vivax*, 15 days for *Plasmodium falciparum*, and 25 days for *Plasmodium malariae* [54]. In the end, risk analysis was limited to human infections with *Plasmodium falciparum*, the only plasmodia species responsible for fatal cases [6].

**Fig 4:**
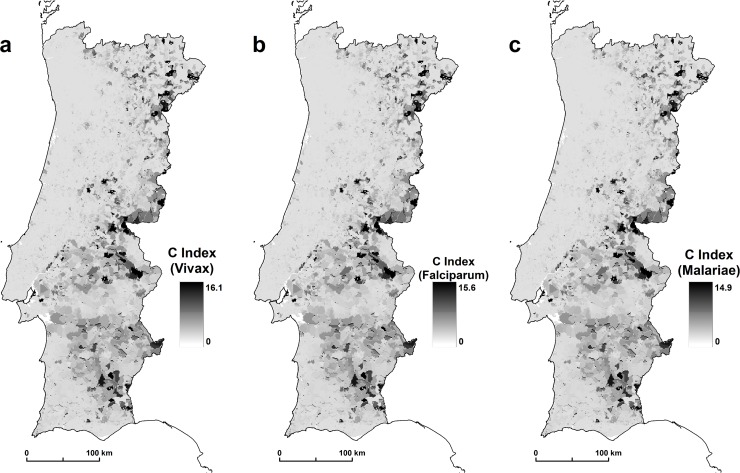
(*C—receptivity*) for *An*. *atroparvus* (a) *Plasmodium vivax*, (b) *Plasmodium falciparum*, and (c) *Plasmodium malariae*.

The results show that *An*. *atroparvus* has a variable capacity to transmit malaria. The results of the three scenarios show the incubation period of 16.1 days for *Plasmodium vivax*, 15.6 for *Plasmodium falciparum*, and 14.9 for *Plasmodium malariae*. *Plasmodium vivax* is a greater causative factor because this species, under the same environmental conditions as other species, has a shorter incubation period. Finally, after calculating all the receptivity components of *An*. *atropravus*, we calculated the *C* index. The scale of the values retrieved must be interpreted in a qualitative way, where the areas with higher values correspond to areas of larger receptivity and the areas with lower values must be understood as having less receptivity to disease emergence (Fig 4).

### Infectivity

Infectivity of mosquitoes in humans, also known as vector competence, estimates the proportion of female mosquitoes that become infective after a blood meal from an infected person. This value was determined from the work of [9]. Colony specimens of *An*. *atroparvus* were artificially infected with tropical strains of *Plasmodium falciparum* (*i*.*e*. NF54 and NF60). Although considered a low competence vector, results show that the infectivity of this species can reach 0.135, depending on environmental conditions and the nutritional status of the female mosquito.

### Vulnerability

Regional vulnerability is the proportion of infected humans with forms of the parasite that are also infective in mosquitoes. For the infected humans, we used for the A0 scenario the imported cases of malaria cases for the year 2013 [55].

The malaria patients diagnosed in Portugal was an significant increase from 2010 to 2013: 54 imported cases in 2010 to 123 imported cases in 2013 (Table 4).

**Table 4:** Imported malaria cases in Portugal, from 2010 to 2013 [55].

	2010	2011	2012	2013
**Imported malaria cases**	54	64	58	123

Currently, the reported cases of malaria in Portugal are underestimated [56]. Therefore, in order to minimize this gap, and to come as close to reality as possible, we introduced scenarios A1 and A2. In these scenarios, we increased the number of infected people: 200 for scenario A1, and 400 for scenario A2. These infected people, for each model, were introduced randomly at municipality level.

## Discussion and Results

**Fig 5:**
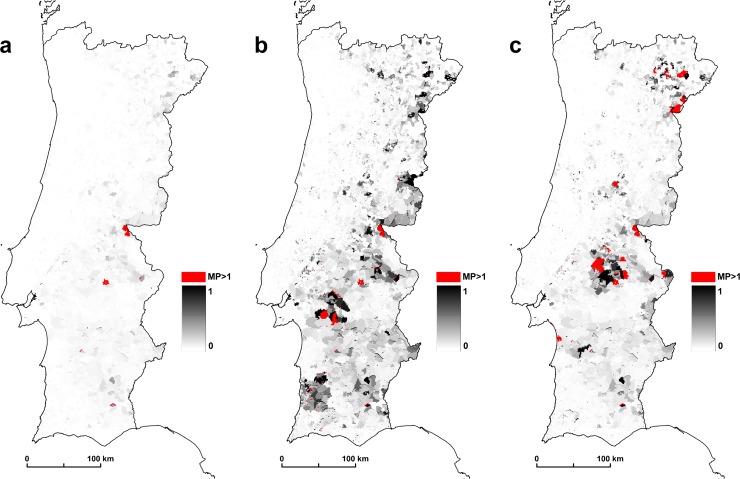
(a) scenario A0, (b) scenario A1, and (c) scenario A2.

Our results suggest the higher and lower risk areas of malaria resurgence in mainland Portugal. We consider as hotspots the areas > 1. In scenario A0, we identified a total area of 134 km^2^ with values >1; in A1, 351 km^2^; and in scenario A2, we identified 847 km^2^. These results show the importance of the imported cases of malaria cases in the simulation for the appearance of hotspots. All these areas are the high risk areas of malaria resurgence. At the national level, the results showed that there are spatial variations in the risk of malaria transmission. The areas of greatest risk are in northern inland areas, in the upper and lower Alentejo region, and throughout the course of the Tagus and Sado Rivers (Fig 5).

**Fig 6:**
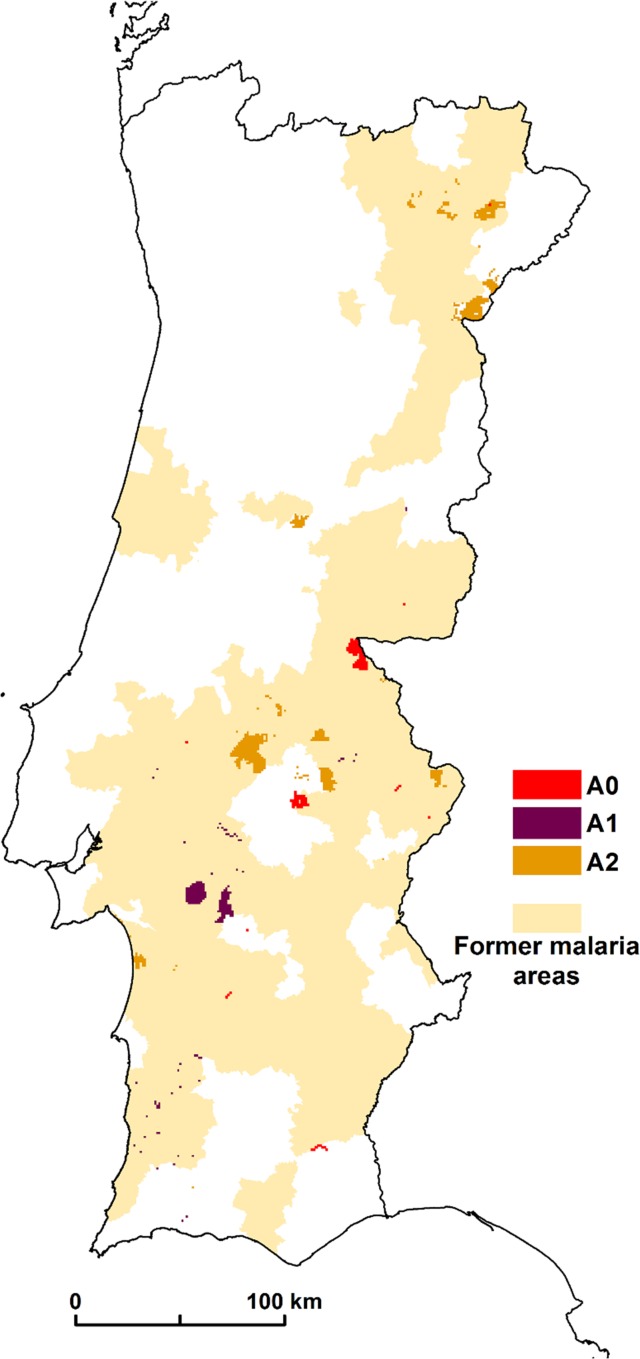
Concordance between former malaria areas and malariogenic potential >1 in scenario A0, A1, and A2.

The spatial distribution patterns of malaria in Portugal were documented by (6). We compared this former malaria distribution with the malariogenic potential >1 for the 3 scenarios. For scenario A0 there are 91.8% of concordance; for A1, 95.1%; and for scenario A2, there are 73.1% of concordance (Fig 6).

These results show a high level of similarity between the former malaria spatial distribution and the hotspots for the 3 scenarios.

## Conclusions

In this study, we identified the areas of greatest potential risk of malaria resurgence. The results obtained demonstrate a clear geographic variation in the risk, which is greatest in the northern interior, the Alentejo region, and along the basins of the Tagus and Sado rivers. These areas are less densely populated [57], which contrasts with areas of lower risk that have a higher population density.

We did identify areas of possible malaria resurgence in some areas in Alentejo and in the northern interior, particularly with the increase of infected people in scenarios A1 and A2. Future work should include more entomological studies on *An*. *atroparvus*, especially with larger temporal and spatial variation and more precision on the location of diagnosed malaria patients (imported cases). The methodology of this work should be an aid to guide health policy in monitoring and preventing potential risks of resurgence of endemic malaria outbreaks.
